# Experimental Study on Impact Performance of Basalt-Polypropylene Fiber Reinforced High-Performance Concrete

**DOI:** 10.3390/ma17133253

**Published:** 2024-07-02

**Authors:** Maoyu Zhang, Bo Li, Zezhong Zheng, Jicheng Zhang

**Affiliations:** 1 School of Civil Engineering and Architecture, Taizhou University, Jiaojiang 318000, China; ryanzmy0577@tzc.edu.cn; 2 Department of Civil Engineering, Wenzhou University of Technology, Wenzhou 325035, China; 3 School of Urban Construction, Yangtze University, Jingzhou 434023, China; 2022710706@yangtzeu.edu.cn (Z.Z.); zhangjicheng@yangtzeu.edu.cn (J.Z.)

**Keywords:** high-performance concrete, basalt fiber, polypropylene fiber, impact, microstructure, Weibull

## Abstract

To enhance the impact resistance of high-performance concrete (HPC), a novel efficient solution was adopted by incorporating basalt fibers (BF) and polypropylene fibers (PF) as reinforcement materials in this work. To this end, the effects of single BF (BHC) and PF (PHC) as well as their combinations (BPHPC) on the impact energy consumption, ductility ratio, and toughness factor were explored through drop weight impact test of concrete considering fiber volume contents (0.1%, 0.15%, 0.2%) to evaluate the impact resistance of the concrete. The Weibull distribution function model is used to fit the drop weight impact test results and predict the probability of failure. Moreover, the fracture-resistance enhancement mechanism of fiber is analyzed at a microscopic level. Test results showed that the number of impacts resisted by the HPC can follow well the two-parameter Weibull distribution. Compared with the single BF and single PF, the combination of 0.15% BF and 0.1% PF yields favorable impact resistance, thus exhibiting a positive hybrid effect.

## 1. Introduction

Fiber reinforced high-performance concrete (FRHPC) is made by adding a certain volume fraction of fibers to the HPC matrix. It has been found that steel fibers, polypropylene fibers, and basalt fibers can improve the compressive and flexural properties of HPC to some extent [1,2,3]. There exists a need to develop cement concrete characterized by enhanced crack resistance and durability. The initiation, propagation, and coalescence of micro- or macro-cracks can be prevented, controlled, and retarded by adding fibers to concrete [4,5,6,7,8,9]. In recent years, FRHPC has been utilized extensively in numerous infrastructure construction projects. As FRHPC is used more frequently, the demands placed on it are increasing. In some cases, FRHPC must withstand impact loads caused by mechanical operations, means of transport, and natural disasters such as earthquakes. Impact load is a crucial element that cannot be ignored throughout the service life of the building.

Most previous studies into the impact properties of FRHPC have focused on steel fibers. For instance, these targets, made of steel fiber reinforced high-performance concrete (SFRHPC) and high-strength concrete, were studied by Luo et al. [10] and subjected to high velocity impacts by projectiles. Test results showed that high-strength concrete targets were shattered, while SFRHPC targets maintained integrity with only a few cracks on their surfaces. The SFRHPC target developed severe cracking and the failure mode became pseudoplastic. Chien et al. [11] carried out static compression and dynamic impact tests on SFRHPC specimens with fly ash by split Hopkinson pressure bar (SHPB). On the premise that the volume fractions of the end hook steel fibers were 0.5%, 1.0%, and 1.5% respectively, the brittle damage of the concrete gradually transformed into ductile damage as the volume fraction of the fibers increased. Song et al. [12] found that the addition of steel fibers to high-strength concrete could significantly improve the impact resistance of concrete through drop hammer impact tests and indicated that the first-crack strength and failure strength of high-strength concrete were approximately normally distributed, while the opposite was true for high-strength steel fiber reinforced concrete. Li et al. [13] pointed out that the impact resistance and flexural toughness are more sensitive to the steel fibers by investigating the synergistic effect of steel fibers and coarse aggregates on the impact behavior of ultrahigh performance fiber reinforced concrete. However, while steel fibers enhance the mechanical attributes of concrete, the susceptibility to corrosion in environments may lead to a decline in inherent properties, and the high cost of steel fiber has prevented it from being used extensively in actual construction projects. To reduce the cost, basalt fiber was selected, which had a performance no lower than that of steel fiber but was less expensive. In experiments, some researchers discovered that adding BF to concrete could give it good mechanical properties. BF is a new inorganic, environmentally friendly, high performance fiber material drawn from natural basalt, which is considered superior to others because of their comparable mechanical strength, higher durability than glass fibers, lower cost than carbon fibers, sustainability due to abundant raw material, and environment-friendly production process. It possessed high elastic modulus and excellent resistance to corrosion, acid and alkaline conditions, and high and low temperatures [14,15,16]. The tensile, flexural, and pre-crack strength of concrete will be enhanced with the use of BF [17,18,19].

However, conventional FRHPC involves only one type of fiber, which is effective in providing strain and crack resistance to a limited extent. Research has proved that hybrid fibers are better than single fiber, particularly the hybridization of rigid fibers (e.g., steel fiber and BF) and flexible fibers (e.g., PF and polyvinyl alcohol fiber) contributes to improving the strength and toughness of concrete [20,21]. Rigid fibers provide initial cracking stress and ultimate strength, while flexible fibers increase toughness and strain in the crack zone [22]. Thus, the advantages of FRHPC can be maximized by hybridizing two or more fiber types [23].

PF can not only prevent drying shrinkage cracks in concrete, but also can enhance the durability and improve the service life of concrete structures due to its chemical inertness [24,25]. Wang et al. [26] investigated the synergistic effect of BF and PF on the compressive strength, flexural strength, and splitting tensile strength of HPC. Test results indicated that the optimum mixing ratio was 0.15% BF and 0.033% PF. Zhang et al. [27] considered that both BF and macro synthetic PF could enhance the impact strength of ordinary concrete. BF is stronger than PF in the impact strength of concrete and weaker than PF in terms of impact toughness resistance. A moderate amount of BF and PF are effective in increasing the strength of concrete, but too much is not worth the cost.

It is known from the literature that the hybridization of BF and PF could be given expectations in terms of improving the strength and toughness of concrete under impact loading. Nonetheless, to the best of the author’s knowledge, the impact resistance of BPHPC had not been studied extensively. Hence, to make up for the research in this field blank, impact tests were carried out in this present study to investigate the HPC beams reinforced with BF and PF. BF and PF were added to the HPC specimens at 0.1%, 0.15%, and 0.2% volume fractions. 100 mm × 100 mm × 400 mm beam samples were cast for impact tests by a self-developed drop-weight testing system. Most studies on the mechanical properties of concrete have been based on macroscopic physical experiments. Thus, in order to be able to study BPHPC from a microscopic perspective, microscopic test results from scanning electron microscopy (SEM) were used to assist in the analysis of the macroscopic mechanical test results. The results of this experiment provide an experimental and theoretical reference for the pervasive application of BPHPC, especially in impact loading environments.

## 2. Experimental Program

### 2.1. Raw Materials

**Figure 1:**
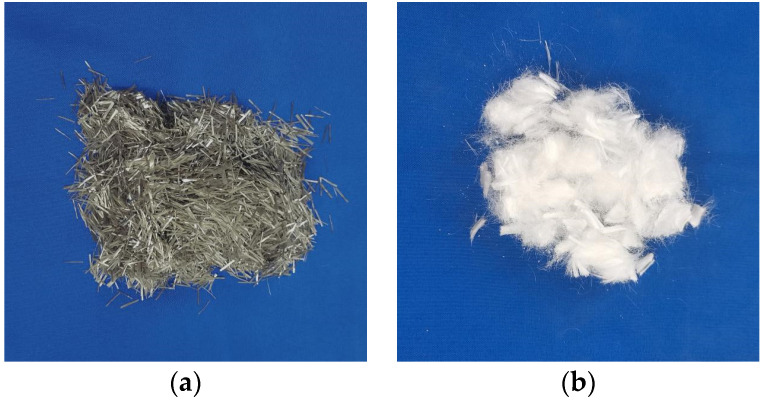
Fiber appearance diagram: (**a**) BF; (**b**) PF.

To prepare the HPC specimens, local ordinary Portland cement (P.O 52.5) was employed herein as the binder; its physical behaviors are listed in Table 1. Silica fume and fine-grade fly ash are crucial components of HPC, which can improve the microstructure and enhance the mechanical properties and durability of HPC [28]; the properties of silica fume and fly ash are given in Table 2 and Table 3, respectively. In addition, concrete will give off a lot of heat when cement hydrates during the preparation process. The fly ash that replaces part of the cement carries out a volcanic ash reaction and generates new cementitious products to fill the concrete voids, thus improving the density of concrete and preventing the concrete from cracking. The silica fume used in this project has a PH value of neutral and a 7-day activity index of 105%. Silica fume can fill the gaps between cement particles, thereby improving the fluidity of concrete. At the same time, the addition of silica fume can reduce the heat release without reducing the strength, and the simultaneous use of silica fume and superplasticizer can save cement and increase the strength of concrete. The fineness modulus of fine aggregate is 2.6, and the particle size of coarse aggregate is 5–10 mm. A polycarboxylate superplasticizer with a water-reducing rate of 30% was used to adjust the workability of HPC. The local drinkable tap water from Jingzhou is used for mixing. The reinforcement materials for HPC are BF and PF, whose mechanical performance parameters of BF and PF are presented in Table 4, and their appearance is shown in Figure 1.

### 2.2. Mix Design of HPC and Fiber Content

The mixture proportions for the concrete matrix are identical whereas the only differences are the type and volume fraction of the fibers. The mixing proportion by weight is 1:0.2:0.13:1.13:2.64:0.03:0.32 (cement:silica fume:fly ash:fine aggregate:coarse aggregate:superplasticizer:water).

The two types of fibers used in the test are non-metallic fibers. When the fiber content is too high, the fibers tend to agglomerate and the weak interfacial effect between the fibers and the concrete becomes more pronounced, which reduces the mechanical properties of HPC. Therefore, the total volume fraction of fibers corresponding to each concrete specimen is below 0.4%. The information on specimens is listed in Table 5. The abbreviations are arranged for specimens in line with the type and content fiber. For instance, B0.1P0.15 indicates that the volume fraction of BF and PF are 0.1% and 0.15%, respectively.

### 2.3. Mixing Procedure and Specimen Preparation

The mixing procedure for HPC was that cement, silica fume, fly ash, fine aggregate, and coarse aggregate were successively added and slowly mixed at a speed of 61.5 rpm for 3 min in a dry state. Then, to evenly disperse the fibers, BF and PF were added into a rotating mixer and mixed again for five minutes. The next step was to mix the water and superplasticizer in a certain proportion, and half of the freshly prepared liquid was poured into the mixer. Subsequently, water and superplasticizer were added into the mixture to further mix until a quasi-liquid state was reached. Then, the paste was rapidly stirred for 2 min at a speed of 123 rpm. Finally, the paste was mixed slowly at a speed of 61.5 rpm for another 1 min [29,30]; the whole process took no more than 15 min. Finally, the mixture that was poured into the mold was slightly vibrated on a high-frequency vibrating table. All the specimens were cured in a laboratory at room temperature for 24 h before demoulding. After that, the molded specimens were cured in water at 20 ± 2 °C for up to 28 days.

### 2.4. Drop Weight Impact Test

**Figure 2:**
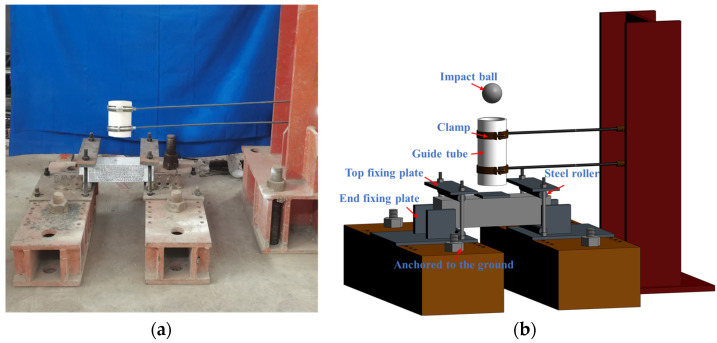
Drop weight impact test device: (**a**) Picture of device; (**b**) Schematic diagram of device.

The drop weight impact test was conducted by using the self-developed impact device in accordance with suggestions of the national standard GB/21120-2018 [31]. The test set-up for the drop weight impact test is illustrated in Figure 2. Ninety-six samples with dimensions of 100 mm × 100 mm × 400 mm were used for impact tests in this study. Before the test, the 100 mm × 100 mm × 400 mm beam samples were placed on the steel rollers with a span length of 300 mm. A 2 kg steel hammer was repeatedly dropped from an initial height of 300 mm on a square steel plate with a side length of 100 mm, which was positioned at the center of the specimen surface. A guide tube was arranged to ensure that the impact ball would land precisely in the center when it fell. To ensure that the pipe was vertical, it was held in place with two adjustable length clamps. The surface cracking and damage of the specimen were carefully observed with a magnifying glass after each impact. As the number of impacts increased, the number of impacts was recorded as *N*_1_ when the first visible crack appeared in the specimen and the crack width reached 0.05 mm. It indicated the number of impacts required for the initial crack to occur and also the initial crack strength. The number of impacts was recorded as *N*_2_ when the main crack penetrated the entire section of the specimen. It was expressed as the number of impacts required to damage the specimen and represented the failure strength. The ability of the specimen to absorb energy and resist deformation after the initial crack appeared could be reflected by the ductility ratio *µ* and the toughness coefficient *C*. Correspondingly, the *µ*, *C*, and impact energy for each specimen can be calculated using the following Equations (1)–(3).

(1) $$\mu =\frac{\left(N_{2}-N_{1}\right)}{N_{1}}$$

(2) $$C=\frac{W_{i}}{W_{0}}$$

(3) $$W_{i}=N_{i}mgh$$

where $W_{i}$ is the impact energy consumption (J); $W_{0}$ is the impact energy consumption of control (J); $N_{i}$ is the number of impacts; *m* is the mass of the impact ball; *g* is gravity acceleration, 9.8 N/kg; *h* is the impact height.

## 3. Results and Discussion

### 3.1. Drop Weight Impact Test Results and Analysis

The drop weight impact test results of this test series are presented in Table 6. The number of impacts at first-crack generation *N*_1_, the number of impacts at damage *N*_2_, the ductility ratio *C*, and the toughness coefficient *µ* are listed.

As can be seen from Table 6, the *N*_1_ and *N*_2_ of the specimens without fibers are almost equal, which indicates that the initial cracking and damage occur almost simultaneously. The specimens without fibers showed obvious brittle characteristics and were damaged more severely under impact loading. In addition, the impact energy dissipation of the specimens increased gradually, regardless of whether a type of fiber multi-fibers was added. For example, after adding BF alone, the B0.1, B0.15, and B0.2 specimens provided 36%, 50%, and 68% more impact energy consumption at damage than the control, respectively. On the other hand, the impact energy consumption of PHPC specimens increased with increasing PF content at damage. The impact energy consumption at the damage was increased by 32%, 50%, and 68% by adding 0.1%, 0.15%, or 0.20% PF into the matrix, respectively. The maximum impact energy consumption of the BPHPC increased by 168% compared to the control. The dispersion and distribution of fibers in HPC form a large number of micro bridges, which can prevent the expansion of cracks in concrete and make it possesses excellent impact resistance performance. As the number of fibers increased, leading to a reduction in fiber-to-fiber spacing and an increase in the number of fibers per unit area, the bond between the fibers and the HPC gradually increased, which was one of the most important factors in increasing impact resistance.

Meanwhile, *C* and *µ* tended to increase in varying degrees with increasing fiber content. In addition, when the same volume fraction (e.g., BF = 0.1 vol.% or PF = 0.1 vol.%) was incorporated, it was found that the impact energy consumption at the damage of the fiber reinforced plain concrete specimens was slightly higher than that of the fiber reinforced high-performance concrete specimens by comparing this experiment results and the existing test results on the impact resistance [32]. As higher strength concrete is more prone to brittle damage than plain concrete, the improvement in impact resistance of fiber to plain concrete is slightly better than that of fiber reinforced high-performance concrete.

There was a substantial improvement over the control specimens when BF and PF were added to the concrete at the same time. Among the BPHPC specimens, the lowest *C* and *µ* values were obtained for B0.1P0.1 specimen with 0.65 and 1.5, respectively. The B0.2P0.2 specimen had the best impact resistance with *C* and *µ* values of 2.11 and 2.68, respectively. The results showed that the impact energy of BPHPC specimens at damage state ranged from 194.04 J to 346.92 J. This can be credited to the positive effect of hybrid fibers in limiting the development of cracks. BF and PF are supplementary to each other in terms of the elastic modulus. PF with low elastic modulus can delay the formation and propagation of micro-cracks in the early stage of hardening and reduce the number of cracks. Crack propagation does little harm to hardened concrete since BF has a high elastic modulus [26]. Accordingly, both BF and PF play an important part in improving the impact resistance of HPC.

### 3.2. Weibull Distribution Model

Many irregular micro-cracks and micro-pores are prevalent within the concrete, which has a multi-phase and inhomogeneous nature, and there remains a large randomness for the test data under the same test conditions. Thus, only a detailed study of its probability distribution can determine its impact resistance pattern more accurately and comprehensively. In the past decades, statistical models such as normal, lognormal, and Weibull, proposed by the American Society for Testing and Materials (ASTM), have been applied in the analysis of concrete fatigue, freeze-thaw damage, and impact test data [33,34,35].

However, normal and log-normal distribution models are less applicable and flexible in reliability analysis applications and do not model the various states of material damage well. Simpler data handling, wider adaptability, better flexibility, smaller data sample sizes, and the ability to give more accurate fault predictions with fewer samples than the normal distribution are features of the Weibull distribution. Therefore, Weibull distribution is widely used in the study of fatigue properties and impact properties of concrete.

Suppose that the number of times *N_x_* that the concrete resists the impact follows the Weibull distribution according to the definition of the Weibull distribution function. The probability density function *f*(*N_x_*) and the cumulative distribution function *F*(*N_x_*) of *N_x_* can be expressed as

(4) $$f\left(N_{x}\right)=\frac{\alpha }{N_{a}-N_{0}}{\left(\frac{N_{x}-N_{0}}{N_{a}-N_{0}}\right)}^{\alpha -1}\exp\left[-{\left(\frac{N_{x}-N_{0}}{N_{a}-N_{0}}\right)}^{\alpha }\right]$$

(5) $$F\left(N_{x}\right)=1-\exp\left[-{\left(\frac{N_{x}-N_{0}}{N_{a}-N_{0}}\right)}^{\alpha }\right]$$

In this equation, *α* represents the Weibull modulus or shape parameter; *N_a_* is the Weibull scale parameter; *N*_0_ is the minimum life parameter; and *N_x_* is the specific value of the random variables (i.e., *N*_1_ and *N*_2_ in this experiment); where *α* > 0; *N_a_* > *N*_0_; *N_x_* ≥ *N*_0_.

The minimum life parameter *N*_0_ = 0 can be assumed for safety, reliability, and computational simplicity, rewrite Equations (4) and (5) as

(6) $$f\left(N_{x}\right)=\frac{\alpha }{N_{a}}{\left(\frac{N_{x}}{N_{a}}\right)}^{\alpha -1}\exp\left[-{\left(\frac{N_{x}}{N_{a}}\right)}^{\alpha }\right]$$

(7) $$F\left(N_{x}\right)=1-\exp\left[-{\left(\frac{N_{x}}{N_{a}}\right)}^{\alpha }\right]$$

The cumulative distribution function *F*(*N_x_*) represents the probability that the number of impacts is less than a certain observed value, which corresponds to the failure probability function of the specimen, and conversely, the survival probability *P*(*N_x_*) for a specimen with the number of impacts greater than a certain observed value *N_x_* is given by

(8) $$P\left(N_{x}\right)=1-F\left(N_{x}\right)=\exp\left[-{\left(\frac{N_{x}}{N_{a}}\right)}^{\alpha }\right]$$

Taking logarithms twice in both sides of Equation (8) and rearrange this equation, which can be written as

(9) $$ln\left[ln\left(\frac{1}{P\left(N_{x}\right)}\right)\right]=\alpha lnN_{x}-\alpha lnN_{a}$$

Substituting Y =ln[ln(1/P(Nx))], $X\;=\ln(N_{x})$, β = αlnNx into Equation (9) to obtain *Y* = *αX* − *β*. Obviously, the approximate linear relationship exists between *X* and *Y*. The parameters *α*, *β* and the correlation coefficient *R*^2^ are obtained by linear regression analysis. To obtain a graphically tractable expression in terms of *P*(*N_x_*), the number of impacts resisted by each group of specimens is first arranged in increasing order, and then the result is given

(10) $$P\left(N_{x}\right)=1-\frac{i-0.3}{n+0.4}$$

where *n* is the total number of samples per group of specimens and i is the failure order number, i = 1, 2, 3, …, *n*.

**Figure 3:**
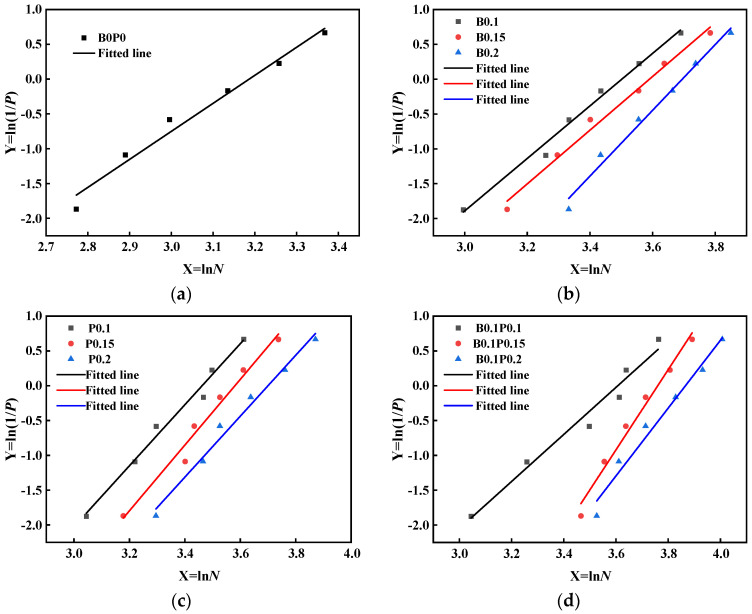

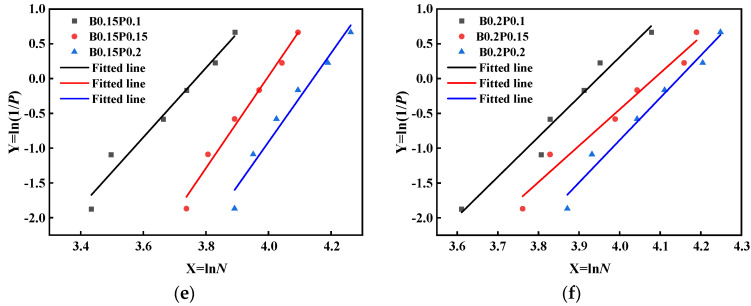
Linear regression curves of *N*_2_ in Weibull distribution: (**a**) HPC; (**b**) HPC with BF; (**c**) HPC with PF; (**d**) HPC with 0.1BF and PF; (**e**) HPC with 0.15BFand PF; (**f**) HPC with 0.2BF and PF.

According to Equations (9) and (10), the Weibull distribution fit test is performed for *N*_1_ and *N*_2_ of all specimens in conjunction with the data in Table 7 with *X* as the horizontal coordinate and *Y* as the vertical coordinate. The linear regression analysis of the experimental data is performed by software, and the regression results corresponding to the regression plots are obtained, as shown in Figure 3 and Table 7, with an intercept of ln *N_a_* and the slope of *α*. *N*_1_ and *N*_2_ for all specimens are used for the linear fit, with a minimum correlation coefficient *R*^2^ of 0.839 and a maximum value of 0.996, with most of the data close to 1. Rahmani et al. [35] discovered that *X* was linearly correlated with *Y* when the *R*^2^ was within the range of 0.7–1.0 through investigation. From Figure 3, the sample values are roughly distributed in a straight line. Consequently, it can be concluded that a two-parameter Weibull distribution is feasible for describing the statistical characteristics of failure strength in the drop weight impact test.

### 3.3. Impact Life Estimation

Based on Equation (9) and the defined parameters tabulated in Table 4, that is, *α* and *N_a_*, the failure strength corresponding to different failure probabilities *P_r_* (Pr =1 - P(Nx)) can be given in equation as follows:

(11) $$\begin{array}{ll} N_{x}=\exp\left\{\frac{\ln\left[\ln\frac{1}{P\left(N_{x}\right)}\right]+\alpha \ln N_{a}}{\alpha }\right\} & =\exp\left\{\frac{\ln\left[\ln\frac{1}{1-P_{r}}\right]+\beta }{\alpha }\right\} \end{array}$$

This reliability equation allows rapid estimation of failure strength without additional costs and impact tests.

**Figure 4:**
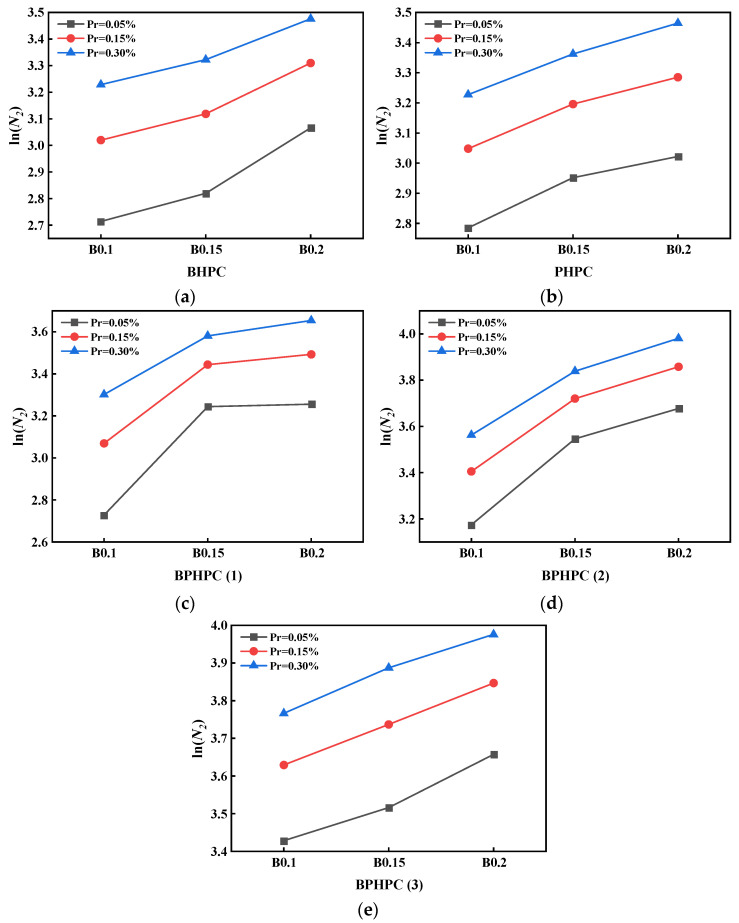
Linear regression curves of *N*_2_ in Weibull distribution of HPC: (**a**) HPC with BF; (**b**) HPC with PF; (**c**) HPC with 0.1BF and PF; (**d**) HPC with 0.15BFand PF; (**e**) HPC with 0.2BF and PF.

In this test, three failure probabilities of 5%, 15%, and 30% are selected for life evaluation, and the *N*_1_ and *N*_2_ of HPC with different failure probabilities can be found according to Equation (11), as shown in Table 8. The line graphs of impact life at different failure probabilities are plotted, as shown in Figure 4. Due to the small *N*_1_ of each specimen, the difference in the number of impacts is not large under different failure probabilities. The *N*_2_ increases with increasing probability of failure, which is consistent with the physical properties of the concrete material. In addition, it is found from Figure 4 that there is an approximately linear correlation between *N*_2_ of HPC and fiber content at different failure probabilities, and the number of impact resistance tends to increase with increasing BF content. The maximum number of impact resistance is reached when the fiber volume content is 0.2%, indicating that BF can effectively improve the impact life of HPC, which is consistent with the experimental results.

### 3.4. SEM Results and Analysis

To observe the microstructure and surface morphology between HPC and fibers and to understand the microscopic characteristics of concrete fracture, damage, and cracks, SEM was utilized to observe the HPC. The surface of the sample was scanned by electron beam to obtain pictures of the microstructure, which was achieved with a TESCAAN MIRA3 digital scanning electron microscope manufactured by TESCAAN Co., Ltd., Brno, Czech Republic, Europe. The samples prepared for testing were as thin as possible and no more than 10 mm in diameter. To explain the effect of fibers on HPC more comprehensively, it was important that the samples prepared contained fibers. The selected SEM samples were glued to a carrier table with conductive tape to hold them in place. Subsequently, the samples were placed in a vent, which not only dried the sample but also blew away any surface impurities. As a final step, the SEM test was carried out after samples were sprayed with a gold conductive coating.

**Figure 5:**
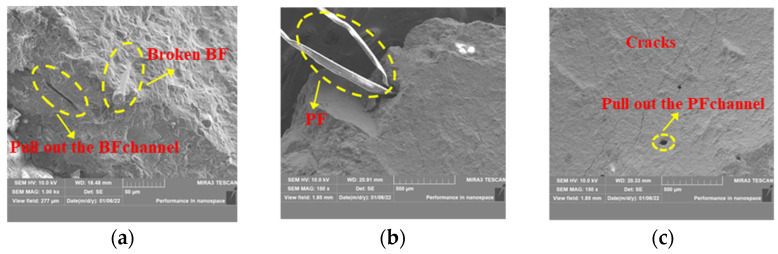
SEM micrographs of BPHPC specimen: (**a**) Micrograph 1 of BPHPC specimen; (**b**) Micrograph 2 of BPHPC specimen; (**c**) Micrograph 3 of BPHPC specimen.

Fibers not only compensate for cracks in compressive tests but also effectively improve the performance of concrete under impact loading. The bonding interfaces of the BPHPC specimens are illustrated in Figure 5. It is observed that the fibers are presented as monofilaments in the concrete and that the cement hydration products are attached to the surfaces of the BF and PF, which can be inferred that the fibers are well bonded to the cement matrix. Many tiny cracks can develop within the concrete when the HPC is impacted. These tiny cracks are bridged by the disorderly distributed fibers, which keeps BPHPC from ‘one-shot’ on impact. Furthermore, the high tensile strength and good binding properties of the fibers chosen mean that the fibers need to absorb a significant amount of energy when they are broken or pulled out. As the stress on the fibers passing through the cracks increases until the stress reaches the bond strength, the fibers are eventually pulled out or pulled off.

However, too many fibers have a tendency to agglomerate into clumps; the fibers on the exterior of the clump can adhere to the cement paste, but the fibers within the clump find it challenging to do so. Due to the lack of compactness, cracks tend to develop within the concrete when it is impacted. In severe cases, the cracks can penetrate right through the entire sample and reduce the mechanical properties of the concrete.

## 4. Conclusions

In this study, the impact resistance of HPC with different fiber contents was investigated. The microstructure characteristics of BPHPC material were observed and analyzed as well. The hybrid effect of the BF and PF on the HPC was evaluated. The conclusions can be drawn as follows:

(1) The impact energy dissipation of the specimens increases gradually regardless of whether a type of fiber or multi-fibers are added. There is little difference in impact resistance between the PHPC specimens and the BPHPC specimens at the same fiber content. HPC with 0.2 vol.% BF and 0.2 vol.% PF showed the best ductility and toughness and the most significant improvement in impact energy dissipation of the specimens with an increase of 168%.
(2) The ability of the BHPC and PHPC specimens to absorb energy and resist deformation is enhanced compared to the control specimens. The maximum values for *C* and *µ*, representing the toughness index of concrete, are obtained from the BPHPC specimens, being 2.11 and 2.68, respectively. In addition, the probability distribution of *N*_2_ for the HPC specimens obeys well the two-parameter Weibull distribution. The *N*_2_ increases as the probability of failure increases. The calculated values of *N*_2_ for specimens with hybrid fibers are greater than those for specimens with single fibers at different probabilities of failure.
(3) BF and PF have a synergistic effect when mixed, complementing each other and acting as a crack arrestor at different structural levels and at different loading stages. This effectively improves the impact damage resistance of HPC, which in turn exhibits better impact resistance than concrete without fibers and concrete with the addition of one type of fiber.

## Figures and Tables

**Table 1:** Main performance parameters of cement.

Cement Grade	P.O 52.5			
Specific surface area (m^2^/kg)	350	Compressive strength (MPa)	3 d	29.2
Specific gravity (g/cm^3^)	3.26		28 d	58.1
Initial setting time (min)	160	Flexural strength (MPa)	3 d	6.8
Final setting time (min)	220		28 d	12.6

**Table 2:** Chemical composition of silica fume.

Composition	SiO_2_	Al_2_O_3_	Fe_2_O_3_	MgO	Na_2_O	CaO
Content (%)	96.16	0.30	0.44	0.29	0.08	0.03

**Table 3:** Performance index of fly ash.

Water Demand Ratio (%)	Loss on Ignition (%)	Moisture Content (%)	SO_3_ (%)	f-CaO (%)
92.8	0.43	0.01	0.44	0.29

**Table 4:** Physical properties of BF and PF.

Fiber Type	Diameter: μm	Fiber Length: mm	Density: g/cm^3^	Modulus of Elasticity: GPa	Tensile Strength: MPa	Fracture Elongation: %
BF	15	12	2.65	93.1–110	3800–4800	3.1
PF	48	12	0.91	3.6	2976	-

**Table 5:** Volume fraction of fiber in HPC.

Types	Specimens	BF (%)	PF (%)	Total Ratio of Fibers (%)
Control	B0P0	-	-	-
BHPC *	B0.1	0.10	-	0.10
	B0.15	0.15	-	0.15
	B0.2	0.20	-	0.20
PHPC *	P0.1	-	0.10	0.10
	P0.15	-	0.15	0.15
	P0.2	-	0.20	0.20
BPHPC	B0.1P0.1	0.10	0.10	0.20
	B0.1P0.15	0.10	0.15	0.25
	B0.1P0.2	0.10	0.20	0.30
	B0.15P0.1	0.15	0.10	0.25
	B0.15P0.15	0.15	0.15	0.30
	B0.15P0.2	0.15	0.20	0.35
	B0.2P0.1	0.20	0.10	0.30
	B0.2P0.15	0.20	0.15	0.35
	B0.2P0.2	0.20	0.20	0.10

* Note: BHPC means that the concrete contains only BF; PHPC means that only PF are added to HPC.

**Table 6:** The impact resistance test results of HPC.

Specimens	*N*_1_	*N*_2_	First-Crack Impact Energy: J	Percentage Increase: %	Impact Energy at Damage: J	Percentage Increase: %	Ductility Ratio *C*	Toughness Coefficient *µ*
B0P0	20	22	117.60	-	129.36	-	0.10	-
B0.1	21	30	123.48	5%	176.40	36%	0.43	1.36
B0.15	24	33	141.12	20%	194.04	50%	0.38	1.50
B0.2	25	37	147.00	25%	217.56	68%	0.48	1.68
P0.1	20	29	117.60	0%	170.52	32%	0.45	1.32
P0.15	19	33	111.72	−5%	194.04	50%	0.74	1.50
P0.2	15	37	88.20	−25%	217.56	68%	1.47	1.68
B0.1P0.1	20	33	117.60	0%	194.04	50%	0.65	1.50
B0.1P0.15	20	40	117.60	0%	235.20	82%	1.00	1.82
B0.1P0.2	18	44	105.84	−10%	258.72	100%	1.44	2.00
B0.15P0.1	22	40	129.36	10%	235.20	82%	0.82	1.82
B0.15P0.15	21	51	123.48	5%	299.88	132%	1.43	2.32
B0.15P0.2	21	59	123.48	5%	346.92	168%	1.81	2.68
B0.2P0.1	22	48	129.36	10%	282.24	118%	1.18	2.18
B0.2P0.15	21	55	123.48	5%	323.40	150%	1.62	2.50
B0.2P0.2	19	59	111.72	−5%	346.92	168%	2.11	2.68

**Table 7:** Results of linear regression of *N*_2_ in Weibull distribution.

Types	Specimens	*C*		
		*α*	*β*	*R* ^2^
HPC	B0P0	4.02743	12.83295	0.97713
BHPC	B0.1	3.77034	13.20289	0.98953
	B0.15	3.85389	13.83538	0.98881
	B0.2	4.72275	17.44962	0.98658
PHPC	P0.1	4.38559	15.18442	0.98512
	P0.15	4.72178	16.90726	0.97802
	P0.2	4.37930	16.20379	0.98417
BPHPC	B0.1P0.1	3.37835	12.18561	0.97163
	B0.1P0.15	5.75300	21.63118	0.98221
	B0.1P0.2	4.86590	18.81239	0.97489
	B0.15P0.1	4.98917	18.80604	0.97075
	B0.15P0.15	6.61403	26.42230	0.97878
	B0.15P0.2	6.38131	26.43576	0.96349
	B0.2P0.1	5.73299	22.62419	0.96630
	B0.2P0.15	5.22391	21.33890	0.96605
	B0.2P0.2	6.08715	25.23375	0.97433

**Table 8:** The number of times the HPC resists impact under different failure probabilities.

Types	Specimens	*P_r_*		
		5%	15%	30%
HPC	B0P0	12	15	19
BHPC	B0.1	15	20	25
	B0.15	17	23	28
	B0.2	21	27	32
PHPC	P0.1	16	21	25
	P0.15	19	24	29
	P0.2	21	27	32
BPHPC	B0.1P0.1	15	22	27
	B0.1P0.15	26	31	36
	B0.1P0.2	26	33	39
	B0.15P0.1	24	30	35
	B0.15P0.15	35	41	46
	B0.15P0.2	40	47	54
	B0.2P0.1	31	38	43
	B0.2P0.15	34	42	49
	B0.2P0.2	39	47	53

## Data Availability

Data are contained within the article.

## Abbreviations

*α*	shape parameter
*β*	intercept
*µ*	ductility ratio
*C*	toughness coefficient
*f*(*N_x_*)	probability density function
*F*(*N_x_*)	cumulative distribution function of *N_x_*
*g*	gravity acceleration
*h*	impact height
*i*	failure order number, i = 1, 2, 3, …, *n*.
*m*	mass of the impact ball
*n*	total number of samples per group of specimens
*N_i_*	number of impacts
*N_x_*	specific value of the random variables (*N*_1_ and *N*_2_ in this experiment)
*N*_0_	minimum life parameter
*N_a_*	scale parameter
*N*_1_	number of impacts at first-crack generation
*N*_2_	number of impacts at damage
*P_r_*	failure probabilities
*P*(*N_x_*)	survival probability
*R* ^2^	correlation coefficients
*W_i_*	impact energy consumption
*W*_0_	impact energy consumption of the control specimen
*X*	horizontal coordinate
*Y*	vertical coordinate
